# Two‐Year Follow‐Up of a Communication Intervention on Medication Adherence and Health Literacy in Kidney Transplanted Recipients—A Randomised Controlled Study

**DOI:** 10.1111/jorc.70010

**Published:** 2025-02-11

**Authors:** Tone K. Vidnes, Astrid K. Wahl, Marie H. Larsen, Käthe B. Meyer, Åsmund Hermansen, Marit H. Andersen

**Affiliations:** ^1^ Department of Transplantation Medicine Oslo University Hospital Oslo Norway; ^2^ Department of Interdisciplinary Health Sciences University of Oslo Oslo Norway; ^3^ Lovisenberg Diaconal University College Oslo Norway; ^4^ Faculty of Social Sciences, Department of Social Work, Child Welfare and Social Policy Oslo Metropolitan University Oslo Norway

**Keywords:** health literacy, kidney transplanted recipients, knowledge translation, long‐term follow‐up, medication adherence

## Abstract

**Background:**

Patients with chronic conditions, including kidney transplanted recipients, are required to actively participate in their continuous care and maintain motivation to adhere consistently to treatment.

**Objectives:**

Our study aimed to test long‐term effectiveness of a new health communication intervention designed to improve on medication adherence and health literacy in kidney transplant recipients 2 years following transplantation.

**Design:**

A randomised controlled non‐blinded study was conducted between March 2020 and August 2023.

**Participants:**

One hundred and ninety‐five kidney transplant recipients were included.

**Measurements:**

Primary outcomes were self‐reported medication adherence, measured by the BAASIS questionnaire, and health literacy, measured by the Health Literacy Questionnaire. The response rate was 87% (170 of 195 patients).

**Results:**

This study showed a significant difference between groups in favour of the intervention group for medication adherence (*p* < 0.02) and two essential Health Literacy Questionnaire domains—‘navigating the health care system’ (*p* < 0.02) and ‘having social support for health’ (*p* > 0.03)—2 years after transplantation. Regarding health literacy, three Health Literacy Questionnaire domains showed a significant correlation with adherence: ‘having sufficient information to manage health’ (*p* < 0.04), ‘having social support for health’ (*p* < 0.04), and ‘ability to understand health information well enough to know what to do’ (*p* < 0.05).

**Conclusion:**

The findings in the study highlight the pivotal role of health communication in enhancing medication adherence and supporting important health literacy aspects for kidney transplant recipients.

**Abbreviations**
HL: health literacy
KTR: kidney transplant recipients

## 1 Introduction

It is essential that health care personnel strengthen kidney transplant recipients (KTRs) in following the medication regimen prescribed for immunosuppressive medication (Gandolfini et al. 2022). Medication adherence is defined as ‘the process by which patients take their medications as prescribed’ (Vrijens et al. 2012, p. 696). Preventing barriers to adherence to immunosuppressive medication in the transplantation population is of outmost importance to decrease the incidence of chronic rejection, donor‐specific antibodies and potential graft loss (Gandolfini et al. 2022). The Norwegian population of KTRs have previously reported nonadherence as high as 38% using the self‐reporting questionnaire BAASIS (Gustavsen et al. 2019). Other studies have reported similar or even higher rates of nonadherence (Lennerling and Forsberg 2012; Scheel et al. 2018). All KTRs in Norway go through an education program during the first few weeks posttransplant that highlights the importance of immunosuppressive medication (Urstad et al. 2013). However, previous research by the same research group has demonstrated that although this education is tailored to each patients' knowledge, their competence when it comes to finding, understanding and using health information, known as health literacy (HL) (Van Der Heide et al. 2018), presents a challenge (Dahl et al. 2020). Health care professionals have an important role in supporting patients with chronic conditions and possibly influencing their HL (Dinh et al. 2022). Further on, HL has been associated with treatment and medication adherence (Hyvert et al. 2023; Stømer et al. 2020), and as self‐reported nonadherence seems to be a frequent problem within the KTR population, it is important to develop and test interventions that improve important patient outcomes such as adherence and HL (Demian et al. 2016).

## 2 Literature Review

KTRs must follow advice, not only regarding immunosuppressive medication (Denhaerynck et al. 2007), but also regarding lifestyle choices such as nutrition, skincare and exercise. Adhering to these recommendations can help delay the long‐term side effects of the immunosuppressive medication and contribute to the success of the transplantation (Jamieson et al. 2016). HL, which is defined by the World Health Organisation as a person's ability ‘to access, understand, appraise, remember and use information about health and health care, for the health and well‐being of themselves and those around them’ (World Health Organisation 2022, p. 14), is of great importance for managing a chronic condition such as being a KTR (Van Der Heide et al. 2018).

HL is a concept that influences what decisions we make in both everyday life and regarding our health (Nutbeam and Muscat 2020). Interventions developed to increase patients' HL often focus on individual attributes; however, it is also important to include the interaction between the individual and health care professionals (Dinh et al. 2022; Van Der Heide et al. 2018). HL research has pointed out the need for interventions to include not only the functional part of HL, but also its interactive and critical aspects (Larsen et al. 2022; Rajah et al. 2018). The intervention evaluated in the present study aimed to strengthen the patient' role as an active knowledge actor using strategies including knowledge translation and motivational interviewing. The intervention had a significant effect on HL change score at 12 months posttransplantation but no effect on medication adherence (Vidnes et al. 2024a). There are some studies showing impact on HL (Visscher et al. 2018) and medication adherence (Torres‐Robles et al. 2018) after interventions. However, studies with long‐term follow‐up on medication adherence and HL in chronic conditions are lacking, which further underscores the importance of such research (Wiecek et al. 2019).

The aim of the current study was to test the long‐term effectiveness of a new health communication intervention on medication adherence and health literacy in KTRs 2 years following transplantation.

## 3 Materials and Methods

### 3.1 Study Design

**Figure 1:**
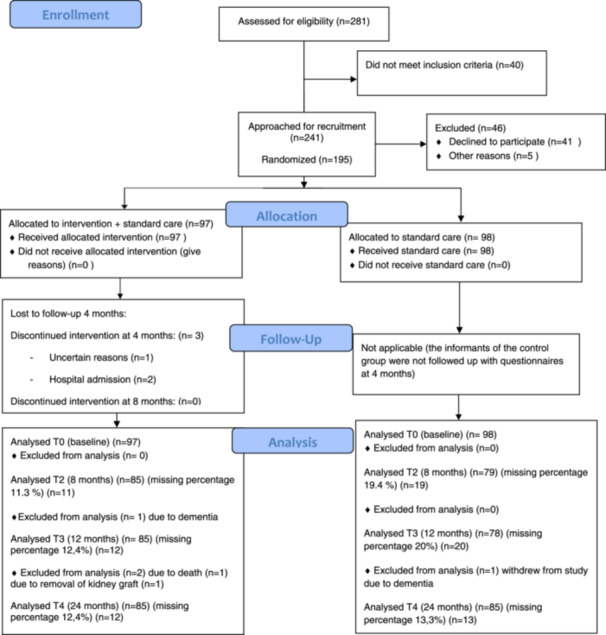
Flow diagram.

A randomised controlled non‐blinded study was performed between March 2020 and August 2023. The study was performed in accordance with CONSORT guidelines; more details are presented elsewhere (Vidnes et al. 2024a). Enrollment is presented in the flow diagram in Figure 1. Patient characteristics and additional descriptive outcomes are presented in Table 1.

**Table 1:** Demographic, clinical and knowledge variables for KTR at baseline and 2 years.

	Intervention (*n* = 97)	Control group (*n* = 98)	*p*‐value
Age at transplantation, mean (years) (SD)	55.4 (13.7)	52.8 (14.3)	0.207^a^
Male (*n* = 114) (%)	56 (58)	58 (59)	0.837^b^
Civil status (*n* = 194)			
Married	73 (75)	61 (62)	0.146^b^
Single/divorced/widowed	7 (7)	9 (9)	
Living with someone	3 (3)	10 (10)	
Living alone	14 (14)	17 (17)	
Other native language than Norwegian (8% in total)	4	12	
Education (university/college) (*n* = 194)	49 (50)	44 (45)	0.628^b^
Work (*n* = 193) (%)	42 (43)	32 (33)	0.038^b^,*
Duration of kidney disease (years) (SD)	16.9 (15.5) (*n* = 85)	15.4 (13.5) (*n* = 86)	0.509^a^
Dialysis treatment (*n* = 192)	66	71	0.732^b^
Time on dialysis (weeks)	101.5 (90.5) (*n* = 66)	105.2 (90.3) (*n* = 67)	0.813^a^
Deceased donor (DD) (*n* = 195)	63 (65)	59 (60)	0.494^b^
Hopkins symptom checklist (SCL‐5) (*n* = 192) (mean baseline) (SD)	1.89 (0.8)	1.88 (0.8)	0.451^a^
Hopkins symptom checlist (SCL‐5) (*n* = 171) (mean 24 months) (SD)	1.78	1.89	0.4^a^
Kidney school at local hospital pre transplantation (*n* = 193)	50 (50)	54 (54)	0.731^b^
Knowledge transplantation (*n* = 195) questionnaire (mean baseline) (SD)	8.9 (2.7)	8.9 (3.3)	0.963^a^
Knowledge transplantation (*n* = 171) questionnaire (mean 24 months) (SD)	9.2 (2.6)	9.2 (2.5)	0.94^a^
VAS score EQ‐5D mean baseline (*n* = 193) (SD)	55 (19)	59 (19)	
VAS score EQ‐5D mean 24 (months [*n* = 170]) (SD)	75 (16)	73 (18)	
Change score EQ‐5D (SD)	19.3 (22)	13.4 (20)	0.08^a^

^a^ Independent *T*‐test.

^b^ Pearson's chi square test.

* Significant difference between groups, *p* < 0.05.

### 3.2 The Intervention—‘KnowMAP’

KnowMAP (Knowledge MAnagement for renal transplanted Patients) was developed and tested in a feasibility study (Andersen et al. 2021) as recommended in complex interventions (Skivington et al. 2024). It was also tested on whether the intervention had an effect on HL and medication adherence 1 year after transplantation (Vidnes et al. 2024a). This health communication intervention is based on principles of knowledge translation and uses collaborative, patient‐centred forms of communication inspired by motivational interviewing (i.e., open ended questions, reflective listening, summarisation, and positive affirmations) (Miller and Rollnick 2012). Interpersonal communication is an important part of making knowledge translation possible for KTRs (Wahl et al. 2022). If patients' don't perceive information as personally relevant, they might not be receptive to it. Hence, if knowledge translation does not occur, patients will have difficulties managing their health properly, which is necessary for those with chronic conditions like KTR (Wahl et al. 2022).

**Figure 2:**
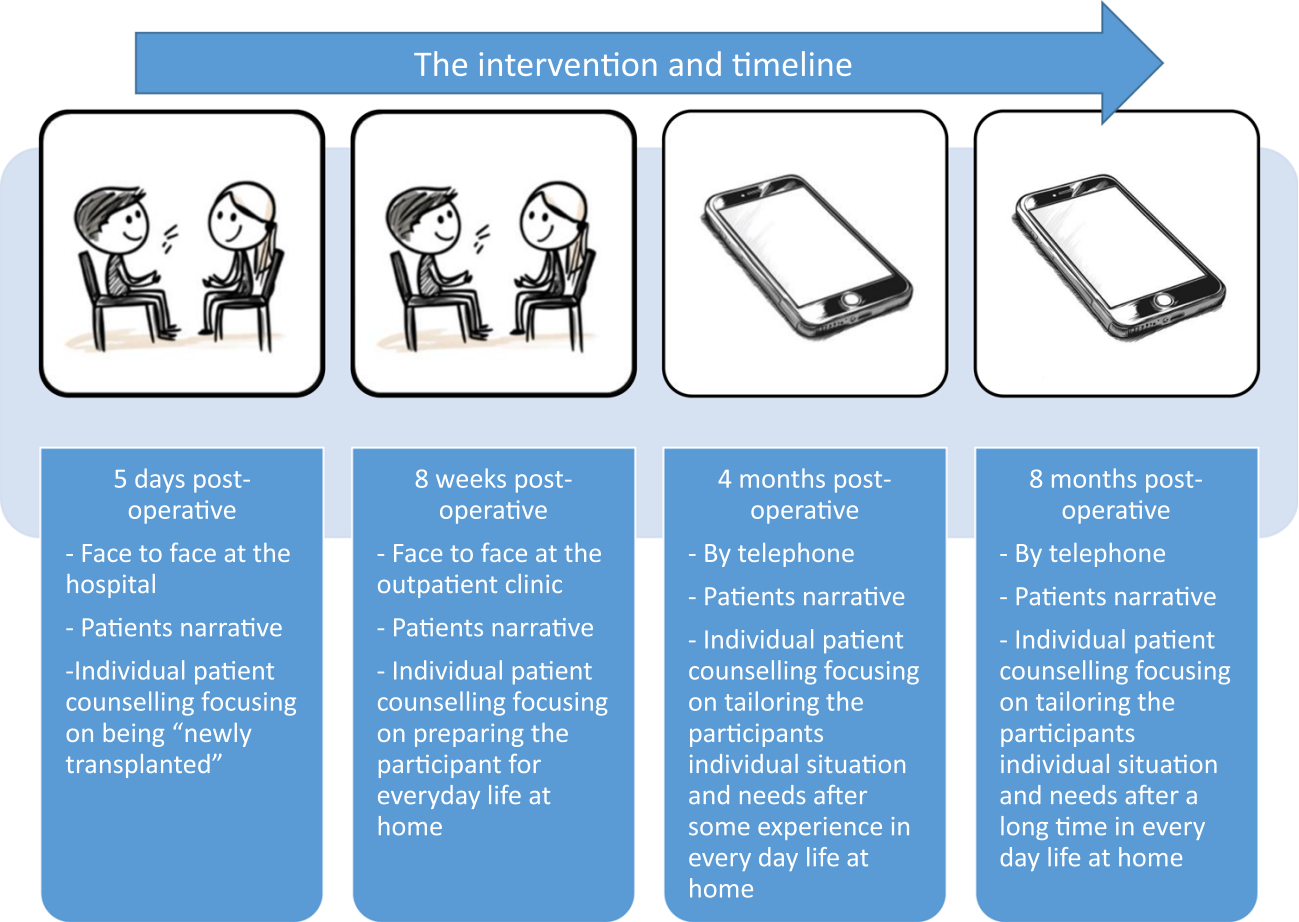
The intervention and timeline.

Following randomisation, patients assigned to the intervention group participated in four communication sessions with a nurse who was trained in the intervention technique (a total of two nurses were trained for this purpose). These sessions took place at specific intervals posttransplantation (5 days, 8 weeks, 4 and 8 months), with the first two being conducted face‐to‐face and the last two by telephone, Figure 2. Each session centred on the patients' narratives, focusing on how they applied the knowledge gained during the posttransplant education in their everyday life and their motivation and barriers for adhering to treatment. Description of topics in the sessions are described elsewhere (Vidnes et al. 2024a). The patients assigned to the intervention group received additional follow‐up with the intervention compared to the patients in the control group.

### 3.3 Sample

Sample size was calculated for the study based on the primary outcome, medication adherence. The assumption that nonadherence could be as much as 50% in the control group was based on research available when planning the study (Denhaerynck et al. 2005). If the intervention reduced nonadherence to 25%, each group would need 58 patients with an *α* of 0.05 and power of 0.80. Earlier research in KTRs reported a 20% drop‐out rate, and due to the 2‐year follow‐up, it was estimated that we would need 100 patients in each group (Tsarpali et al. 2022). During 2020 and 2021, a total of 200 patients were found eligible and 195 patients completed baseline questionnaires. Subsequently, these 195 patients were randomised to a control or an intervention group. The patients were followed for 2 years with questionnaires at different time points, and 170 KTRs filled out the questionnaire at 2 years (87% response rate, Figure 1).

### 3.4 Study Context

The study was conducted at Oslo University Hospital's national transplant centre, which carries out approximately 240 kidney transplants per year and is the sole transplantation facility in Norway.

### 3.5 Data Collection

All eligible patients were presented with oral and written information about the study by nurses in the ward before they were asked to participate, 2–5 days after the transplantation. Informed consent and baseline questionnaires were collected by the nurses in the ward. The nurses responsible for enrolling patients in the study received comprehensive training and were thoroughly briefed on the study's protocols and objectives before its commencement. After completing a baseline questionnaire, the patients were randomised in blocks of 20 with random numbers, into either a control or an intervention group.

- Inclusion criteria: Kidney transplanted ≥ 18 years.
- Exclusion criteria: Kidney transplanted patient in combination with other organs and those with insufficient proficiency in Norwegian to complete questionnaires and carry on a conversation.

After hospital discharge data were collected by the first author (T.K.V.). The 24 month questionnaires were completed using a secure web‐based version provided by the Service for Sensitive Data (TSD). However, 16% of the patients preferred paper questionnaires due to difficulties with computer or email. One reminder was sent to nonresponders by email or telephone.

### 3.6 Primary and Secondary Outcomes

Medication adherence was measured using the self‐reported questionnaire Basel Assessment of Adherence to Immunosuppressive Medication Scale (BAASIS), which measures taking, timing and changing immunosuppressive medication in transplant populations (Dobbels et al. 2010). HL was measured using the multidimensional Health Literacy Questionnaire (HLQ) (Osborne et al. 2013) translated to and validated in Norwegian with good psychometric properties (Urstad et al. 2020). Secondary outcomes were measured with the transplantation knowledge questionnaire (Urstad et al. 2011), the Hopkins Symptoms Checklist (Strand et al. 2003), and the visual analogue scale (VAS) of self‐perceived health status from EQ‐5D (Feng et al. 2020). Graft loss and medication adherence evaluated by the local nephrologist was also taken into account, as registered in the Norwegian National Renal Registry (NNR) (Åsberg et al. 2020).

### 3.7 Instruments

#### 3.7.1 BAASIS and Clinician Score

Adherence to immunosuppressive medication was measured using the self‐reported BAASIS which was developed and is validated for use in the transplant population (Denhaerynck et al. 2023). The questionnaire contains five questions addressing timing, forgetting or changing the dose of immunosuppressive medication. Forgetting, changing or delaying a dose by more than 2 h is classified as nonadherent (Dobbels et al. 2010). The questionnaire has been used in a Norwegian context and found valid and reliable (Gustavsen et al. 2019). At their annual hospital visit, the local clinician scores the KTRs' adherence on a 3‐point scale—poor, suboptimal or excellent—which is registered in the NNR (Åsberg et al. 2020). Only an ‘excellent’ score is classified as adherent; the other two categories are classified as nonadherent.

#### 3.7.2 Health Literacy Questionnaire—HLQ

HL was measured using the Norwegian version of the HLQ questionnaire (Urstad et al. 2020) which consists of 44 questions (two parts) summarised in nine domains. Part 1 (Domains 1–5) consists of questions regarding the patients' self‐reported experiences scored on a scale of 1 (strongly disagree) to 4 (strongly agree). Part 2 (Domains 6–9) consists of questions regarding the patients' capabilities scored from 1 (always difficult) to 5 (always easy). A higher mean score in each domain indicates better HL (Osborne et al. 2013).

#### 3.7.3 Hopkins Symptoms Scale

The Hopkins symptoms checklist (HSCL‐5) questionnaire measures psychological distress using five questions, each with four response options ranging from 1 to 4. A mean score of ≥ 2.0 indicates psychological distress (Schmalbach et al. 2021). The questionnaire has been used and validated in a Norwegian context (Strand et al. 2003) (Table 1).

#### 3.7.4 The Transplantation Knowledge Questionnaire

The transplantation knowledge questionnaire for renal transplant recipients was developed and validated for Norwegian KTRs (Urstad et al. 2011). The questionnaire measures the patients' knowledge in transplantation related‐themes, obtained during an educational program posttransplantation. The questionnaire gives a total score of 1–14 points. A higher score indicates higher knowledge (Table 1).

#### 3.7.5 EQ‐5D—VAS Score

Health status was assessed using the VAS score of the validated EQ‐5D questionnaire. The questionnaire measures self‐reported health status and a total score of self‐perceived health with a VAS score from 0 to 100: the higher the score, the better one's perceived health (Feng et al. 2020; Rabin and Charro 2001) (Table 1). The questionnaire is validated in Norwegian, showing good psychometric properties (Garratt et al. 2021).

### 3.8 Statistical Analysis

All data analyses were based on intention to treat procedure (ITT) (Zelko et al. 2018). StataCorp (2023) and Microsoft Excel 2016 were used for all statistical analysis. Continuous data are presented as means and standard deviations, and categorical data are presented as percentages and frequencies. Medication nonadherence was calculated and analysed using Pearson's chi‐square test and further analysed using logistic regression analysis and reported as average marginal effects (AME). Two‐year change scores in HLQ were calculated and then analysed using *t*‐tests and linear regression analysis. Demographic, clinical and knowledge variables were analysed with Pearson's chi square test and *t*‐test. A *p*‐value of < 0.05 was considered significant. The overall missing responses on all HLQ domains were low, and mean HLQ scores were available for all patients.

### 3.9 Ethical Considerations

The Helsinki Convention guidelines were adhered throughout the research process. Human ethics approval was secured from the hospital's data protection board. Participants received information both verbally and in writing, and informed consent was obtained before their inclusion in the study. Anonymity was maintained by implementing multiple codes within the sensitive data system.

## 4 Results

The main short‐ and long‐term outcomes in the study were medication adherence and HL. Two years after transplantation, a total of 170 patients responded to the follow‐up questionnaires, representing an 87% response rate from the original patients at baseline (*n* = 195).

### 4.1 Medication Adherence

**Figure 3:**
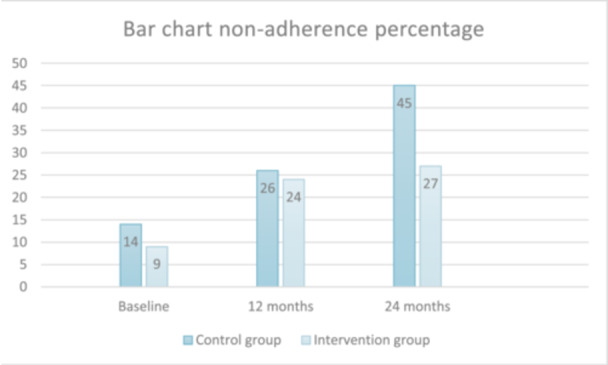
Percentage of nonadherence (BAASIS©).

At 2‐year follow‐up we found a significant difference in adherence between groups, measured with BAASIS, *p* < 0.02 (*n* = 23 in the intervention group and *n* = 38 in the control group reported nonadherence), indicating better medication adherence in the intervention group compared to the control group. Percentage of self‐reported nonadherence (BAASIS) is presented in Figure 3. Further analysis using logistic regression and AME showed that the intervention group had a 17.6 percentage points lower probability of nonadherence compared to the control group.

In addition, data from the NNR demonstrated that only a few patients in the study were classified by their local clinician as nonadherent at 2 years (*n* = 8), and of these eight, only three patients were classified as nonadherent by their local clinician and by the self‐reported questionnaire combined. There was no reporting on graft loss during the second year after transplantation.

### 4.2 Health Literacy

Between‐group effects after 2 years showed statistically significant change scores in 2 HLQ domains in favour of the intervention group. However, only one of the domains from the data at 1 year remained significant. This was Domain 7, reflecting capabilities to navigate the healthcare system (NHS), *p* < 0.02.

The other statistically significant change score in favour of the intervention group was Domain 4, which captures whether patients have social support for health (SS). This domain showed a significant decrease (less social support) in change score from baseline to 2 years in the control group, *p* < 0.04 (Table 2).

**Table 2:** Mean scores and change scores of HLQ at baseline and at 24 months.

HLQ domains	Baseline mean score control group (SD)	24 months mean score control group (SD)	Baseline mean score intervention group (SD)	24 months mean score intervention group (SD)	Control group mean *change score* (SD)	Intervention group mean *change score* (SD)	*Change score p*‐value	*Effect size SRM* (CI)
Domain 1: HPS	3.26 (0.47)	3.11 (0.53)	3.21 (0.44)	3.38 (0.54)	0.35 (0.58)	0.13 (0.57)	0.28	
Domain 2: HSI	3.14 (0.57)	3.14 (0.49)	3.07 (0.53)	3.19 (0.54)	−0.00 (0.52)	0.07 (0.42)	0.33	
Domain 3: AMH	3.16 (0.47)	3.07 (0.41)	3.06 (0.50)	3.11 (0.45)	−0.08 (0.42)	0.02 (0.43)	0.10	
Domain 4: SS	3.26 (0.53)	3.04 (0.56)	3.17 (0.52)	3.13 (0.53)	−0.25 (0.59)	−0.07 (0.49)	**< 0.04**	0.33 (0.63, 0.03)
Domain 5: CA	2.81 (0.56)	2.78 (0.56)	2.62 (0.56)	2.74 (0.59)	−0.03 (0.44)	0.10 (0.52)	0.08	
Domain 6: AE	4.0 (0.58)	3.99 (0.71)	3.9 (0.68)	4.11 (0.65)	−0.03 (0.69)	0.12 (0.65)	0.14	
Domain 7: NHS	3.74 (0.63)	3.70 (0.71)	3.63 (0.71)	3.89 (0.61)	−0.03 (0.75)	0.22 (0.60)	**< 0.02**	0.38 (0.68, 0.07)
Domain 8: FHI	3.73 (0.66)	3.79 (0.65)	3.64 (0.68)	3.83 (0.57)	0.07 (0.62)	0.13 (0.60)	0.53	
Domain 9: UHI	3.88 (0.61)	4.0 (0.55)	3.80 (0.63)	4.10 (0.50)	0.12 (0.51)	0.25 (0.48)	0.10	

*Note:* Baseline, *n* = 19,524 months *n* = 171 change score = 24 months—baseline. *T*‐test mean scores and regression analysis (*p*‐value) effect size (SRM) with (CI).

Abbreviations: AE, ability to actively engage with healthcare providers; AMH, actively managing health; CA, appraise health information; FHI, ability to find good health information; HPS, Feel understood and supported by healthcare providers; HSI, having sufficient information to manage my health; NHS, ability to navigate the healthcare system; SS, have social support for health; UHI, ability to understand health information well enough to know what to do.

Results from the logistic regression, which included the HLQ domains (Table 3), shows that three of the nine domains are significantly associated with adherence in the KTR population. A one‐unit increase in ‘having sufficient information (HSI)’, ‘having social support for health (SS)’, or ‘understanding health information (UHI)’ is associated with an increased likelihood of between 14.3 and 15 percentage points in adherence.

**Table 3:** Health literacy domains and AME.

HLQ variable	AME	*p*‐value
Domain 1—Feel understood and supported by healthcare providers	0.062	0.36
Domain 2—Have sufficient information to manage my health	**0.15**	< 0.04
Domain 3—Actively managing health	0.079	0.34
Domain 4—Have social support for health	**0.145**	< 0.04
Domain 5—Appraise health information	0.007	0.91
Domain 6—Ability to actively engage with healthcare providers	0.020	0.72
Domain 7—Ability to navigate the healthcare system	0.06	0.26
Domain 8—Ability to find health information	0.072	0.23
Domain 9—Ability to understand health information well enough to know what to do	**0.143**	< 0.05

There were no significant findings in the secondary outcomes of this study (Table 1).

## 5 Discussion

The study's 2 year data demonstrated significant difference between groups in terms of medication adherence in favour of the intervention group. Several factors may explain this. First, the patients participated in four sessions where they were invited to speak and were approached in a personalised manner. Second, the intervention began while the patients were in the hospital and felt secure and well taken care of and was implemented over an 8‐month period with four sessions. The first session, entailing individual patient counselling in the ward with a focus on the patient's status as ‘newly transplanted’. The second session took place at an outpatient clinic (8 weeks posttransplant), focusing on preparing the patient for everyday life at home. The third and fourth sessions were both conducted by telephone when the patient was in their home environment and were tailored to the participants' individual situation and needs. Every session during the intervention addressed each patient's unique challenges or barriers to medication adherence and provided them with coaching on what actions to take if needed. Third, the long‐term intervention was performed over a period of 8 months. Lastly, the intervention was led by two nurses with both training in the intervention and expert knowledge of KTRs and medication adherence.

Previous studies have reported nonadherence of 33%–54% in KTRs (Gustavsen et al. 2019; Lennerling and Forsberg 2012; Scheel et al. 2018), using the BAASIS scale. This may indicate that our control group with its 45% nonadherence (38 out of 85) is comparable to earlier studies. However, it is notable that the intervention group, with 23 patients out of 85 (27%) facing barriers to adherence, did not show the same increase from 1 to 2 years. An explanation for this difference could be that the intervention with knowledge translation as a strategy, provided the patients in the intervention group with more knowledge and support about why faithfully taking medication is so important or that these patients developed awareness, which may have provided a tool to help in their everyday life as KTR (Wahl et al. 2022). A meta‐analysis has also reported that it might take time for adherence interventions to reach their potential (Wiecek et al. 2019), which could explain why the difference between groups is significant at 2 years' time. The intervention was conceptualised and refined by a team of researchers that included clinicians, humanists and philosophers and tested in a feasibility study (Andersen et al. 2021), following the recommendations of Skivington (Skivington et al. 2024), and the recommendations of intervening in the context of transplantation (Kostalova et al. 2022). Intervening on patients' personal barriers to medication adherence seems to be useful to support better adherence in the KTR population (Bendersky et al. 2023; De Geest et al. 2021).

Change scores in HL at 2‐year measurement of the HLQ showed a significant decrease in Domain 4 (SS) in the control group (*p* < 0.04). This domain captures having social support for health. Dahl et al. (2020) also found a decrease in Domain 4 (SS) in KTRs within the first 6 months after transplantation (Dahl et al. 2021). Domain 4 (SS) has the ability to make up for the lack of HL in other domains, and the fact that the control group decreased in the domain of social support for health indicates that the KTRs in the control group feel more alone and unsupported in managing their chronic condition (Batterham et al. 2014). While the social networks of transplant patients might perceive them as healthy and no longer needing support, being a KTR is a chronic condition that benefits from ongoing social support for effective self‐management (Memory et al. 2022). Although there is a lack of evidence for how strength in Domain 4 (SS) can make up for in weaknesses in other HL domains, research in self‐management highlights the importance of social support (Chen et al. 2018; Dinh and Bonner 2023).

The other domain that stands out as persistent after testing the new health communication intervention is Domain 7 (NHS), which captures the ability to navigate the health care system. This domain reflects the capability to find help within the health care system and advocate on ones' own behalf at both the system and service level (Osborne et al. 2013). Two years after transplantation, the change score in this domain remains significant in the intervention group (*p* < 0.02) compared to controls, and is even stronger than at the 1‐year measurement (*p* < 0.04) (Vidnes et al. 2024a). Although the effect size is small, the clinical implication may be that the possibility of a supportive dialogue after transplantation strengthens patients' ability to navigate the health care system. Managing a chronic condition as a KTR is a life‐long process (Jamieson et al. 2016; Maasdam et al. 2022). Unnecessary inquiries in the health care system are time consuming and economically unsustainable. The typical KTR has a complex clinical manifestation with comorbidities, often requiring them to navigate several diseases and health care situations (Åsberg et al. 2020). Therefore, it is essential for KTRs to be certain of when and where to seek help in the healthcare system and to be able to advocate on their own behalf when needing health care providers' support (Taylor et al. 2017). The significant change score in Domain 7 (NHS) at the 2‐year mark, indicates that the intervention had a long‐term effect in a domain that is crucial to KTRs for helping them manage their lives posttransplant with all the necessary follow‐up (Grady and Gough 2014). HL responsive health organisations are likely beneficial for patients facing challenges in HL. It is imperative for healthcare systems to develop and implement strategies that are sensitive to patients' age, language and cultural backgrounds, thereby addressing their specific HL requirements (Smith et al. 2024). Additionally, tools designed to support health organisations in becoming more HL responsive are highly relevant (Stømer et al. 2020; Trezona et al. 2018).

Lower levels of HL are seen in association with medication nonadherence (Van Der Gaag et al. 2022). Earlier research using HLQ to measure HL has shown that Domain 4 (SS) is associated with medication adherence (Ostini and Kairuz 2014). This is significant as the intervention group did not show the same decrease in Domain 4 (SS) as the control group did, and there was a significant difference in nonadherence at 2 years. An earlier study using HLQ and self‐reported medication adherence showed that challenges in different HL domains are associated with medication adherence, with Domain 4 (SS) being the most correlated domain (*p* < 0.001) (Demian et al. 2016). Nonadherence is also associated with poorer social support measured with other tools than HLQ (Scheel et al. 2018). Hence, a supportive environment for patients after transplantation might have had an impact on the intervention group. Domain 2, ‘having sufficient information to manage my health (HSI)’ and Domain 9, ‘ability to understand health information well enough to know what to do (UHI)’, were also correlated with nonadherence (Demian et al. 2016); the same domains which showed significant correlations in the current study (Table 3). Hence, the results and correlations observed between nonadherence and HL after testing a health communication intervention indicate that this approach is an important means of enhancing adherence within the KTR population.

## 6 Implicatins for Clinical Practice

Testing a health communication intervention requires patient responsiveness for the intervention to be effective. It demands active involvement and engagement from the patient receiving an intervention for it to make a change as well as the contextual perspective in the intervention (Skivington et al. 2024). The motivational interview technique can stimulate reflectiveness and awareness in individuals (Miller and Rollnick 2012). Transplant experts trained in motivational interview techniques might be beneficial for KTRs. Further on, HL responsiveness organisations could affect the setting in which KTRs receive follow‐up care (Trezona et al. 2018). This is relevant across all levels of the healthcare system, as increasing knowledge regarding HL responsive organisations can improve patient pathways, such as those of KTRs. Enhancements can range from patient information to a more two‐way communication between clinicians and patients and further on facilitating easier navigation of the healthcare system. Particularly concerning post‐transplant comorbidities and conditions (Smith et al. 2024).

The study demonstrated long‐term effects on patients' adherence being of utmost importance for this patient group (De Geest et al. 2021; Skivington et al. 2024). KTRs should be able to interpret and prevent side‐effects of medications over their life course of using immunosuppressive medications, as well as keeping up to date with health information to make informed decisions as a KTRs (Gandolfini et al. 2022). Testing the effects of interventions is well established in health care and self‐management, with HL being a key area of interest (Grijpma et al. 2016; Shao et al. 2023). Patients will have different profiles and different requests according to their preferences and HL needs (Van Der Gaag et al. 2022). It is important to tailor interventions to address these specific needs which could be done by utilising knowledge about patient characteristics that identifies as associated with HL challenges (Vidnes et al. 2024b). Enhancing patients' HL could also positively influence medication adherence.

### 6.1 Strengths and Limitations

The strength of the study is its randomised design including 195 patients, the long‐term follow up, and the high response rate at 2 years (87%). Further, the intervention was performed by just two trained transplant nurses and continuously logged for individual counselling.

Study limitation need to be mentioned. It was not possible to perform a blinded study due to the nature of the intervention. This was a single centre study. Caution should be made when it comes to transferring study results to another context. However, transplantation treatment in Norway is on par with the standards of centres in Northern Europe. Another limitation is that only patients able to understand Norwegian and able to fill out questionnaires were included in the study, possibly leaving those with lowest HL excluded from the study. It is important to be cautious when transferring knowledge from the study to non‐Norwegian speaking KTRs.

## 7 Conclusion

The long‐term results of this randomised controlled study showed a significant difference between groups in favour of the intervention group when it comes to medication adherence and two essential HLQ domains, navigating the health care system and social support for health, 2 years after transplantation. These findings highlight the pivotal role of health communication in enhancing medication adherence and supporting important HL aspects for KTRs.

## Author Contributions

M.H.A. and A.K.W. contributed to study design, T.K.V. performed the study execution and data collection. Å.H., M.H.A., A.K.W., M.H.L., K.B.M. and T.K.V. were all involved in the data analysis or interpretation of the analysis. First manuscript was written by T.K.V. and all co‐authors were involved in and approved the final draft of the manuscript.

## Ethics Statement

Approval was obtained from the Norwegian Ethics Committee for Health Research (#2019/29385). The original study (KnowMAP) was registered as a clinical trial (ClinicalTrials.gov Identifier: NCT04296955). All participants provided written, informed consent.

## Conflicts of Interest

The authors declare no conflicts of interest.

## Data Availability

The data set used and/or analysed during the current study may be available from the corresponding author upon reasonable request.
